# Variations in the quality and costs of end-of-life care, preferences and palliative outcomes for cancer patients by place of death: the QUALYCARE study

**DOI:** 10.1186/1471-2407-10-400

**Published:** 2010-08-02

**Authors:** Barbara Gomes, Paul McCrone, Sue Hall, Jonathan Koffman, Irene J Higginson

**Affiliations:** 1 King's College London, Department of Palliative Care, Policy & Rehabilitation, Cicely Saunders Institute, Bessemer Road, London SE5 9PJ UK; 2 King's College London, Institute of Psychiatry, Box P024 De Crespigny Park, London SE5 8AF UK

## Abstract

**Background:**

Emerging trends and new policies suggest that more cancer patients might die at home in the future. However, not all have equal chances of achieving this. Furthermore, there is lack of evidence to support that those who die at home experience better care and a better death than those who die as inpatients. The QUALYCARE study aims to examine variations in the quality and costs of end-of-life care, preferences and palliative outcomes associated with dying at home or in an institution for cancer patients.

**Methods/Design:**

Mortality followback survey (with a nested case-control study of home vs. hospital deaths) conducted with bereaved relatives of cancer patients in four Primary Care Trusts in London. Potential participants are identified from death registrations and approached by the Office for National Statistics in complete confidence. Data are collected via a postal questionnaire to identify the informal and formal care received in the three months before death and the associated costs, relatives' satisfaction with care, and palliative outcomes for the patients and their relatives. A well-established questionnaire to measure relatives' views on the care integrates four brief and robust tools - the Client Service Receipt Inventory, the Palliative Outcome Scale, the EQ-5 D and the Texas Revised Inventory of Grief. Further questions assess patients and relatives' preferences for place of death. The survey aims to include 500 bereaved relatives (140 who experienced a home death, 205 a hospital death, 115 a hospice death and 40 a nursing home death). Bivariate and multivariate analyses will explore differences in place of death and place of end-of-life care, in preferences for place of death, patients' palliative outcomes and relatives' bereavement outcomes, in relation to place of death. Factors influencing death at home and the costs of end-of-life care by place of death will be identified.

**Discussion:**

Collecting data on end-of-life care retrospectively from bereaved relatives has ethical, practical and scientific challenges. QUALYCARE has been carefully designed to address these challenges in a robust and ethically sound population-based survey. By discovering variations in the underlying individual reality of place of death for people dying from cancer and their families, this study will advance our understanding of end-of-life care and, we hope, improve care for cancer patients and their families in the future.

**Trial registration:**

National Institute of Health Research (NIHR) Clinical Research Network Portfolio. UKCRN7041.

## Background

Extensive evidence shows that most members of the public and patients prefer to be cared for and to die at home [1-3]. However, a recent cross-national study of death certificates showed that only a minority of people dying from cancer achieve this (home deaths ranging from 12.8% in Norway to 45.4% in the Netherlands)[4]. In many countries such as the UK, Italy, Greece, Korea and Japan home deaths have been falling [5-9], but recently in the US and Canada there are indications of a reversal of trends [10-12]. Strategic national decisions were made for Medicare and Medicaid programs in the US and most recently by the Department of Health in the UK to focus on home-based models of care to enable more people to die at home [13,14].

Not all people with terminal cancer have equal chances of dying at home, and some of the inequities are well known. For example, patients living in deprived areas are less likely to die at home. This and another 16 factors are part of a robust international model of factors influencing death at home in cancer, derived from findings of 58 studies relating to 1.5 million patients [15]. This model is in place to guide practice and policy in helping more terminally ill cancer patients to die at home. However, there are variations (individual and geographical) not yet explained.

Enabling more patients to die in the place of their choice is important. However, the real issue is at what quality, and secondly, at what cost. There is little evidence to support that those who die at home and their relatives experience better care than those who die in institutions such as hospitals, hospices or nursing homes. There is a dearth of comparative studies on the associated costs.

The QUALYCARE study has been designed to address the lack of evidence on the quality and costs of end-of-life care for people with cancer in relation to place of death. Its primary aim is to examine variations in the quality and costs of end-of-life care, preferences and palliative outcomes associated with home vs. institutional death in cancer. Secondary aims are to examine whether the care, costs, preferences and outcomes associated with death at home are the same in areas with high vs. low home death rates and in areas with high vs. low deprivation levels. Table 1 details the study objectives.

**Table 1:** Study objectives

**Objective 1**. To describe and compare home, hospital, hospice and nursing home deaths in terms of:
1.1. place where the patients' spent most of the three months before death;
1.2. patients and relatives' preferences for place of death;
1.3. patients' palliative outcomes (symptoms and quality of life in the week before death; attainment of their preferred place of death) and relatives' outcomes (grief intensity; attainment of their preferred place of death);
1.4. use, type and costs of and relatives' satisfaction with formal care received at home and in institutions in the three months before death (including GP care, district nursing, specialist nursing, specialist home palliative care teams; nursing and medical care in hospitals, inpatient hospices and nursing homes);
1.5. use, type and costs of informal care in the three months before death.
**Objective 2**. To describe and compare home vs. institutional deaths on the same five aspects as in objective 1;
**Objective 3**. To describe and compare deaths in high vs. low home death rate areas and high vs. low deprivation levels on the same five aspects as in objective 1;
**Objective 4**. To determine the relative influence of different factors on death at home (vs. in institutions and vs. hospital deaths) and the amount of variation in place of death they explain, comparing areas with high and low home death rates and with high and low deprivation levels;
**Objective 5**. To determine whether death at home (vs. in institutions and vs. hospital deaths) is independently associated with outcomes and costs, comparing areas with high and low home death rates and with high and low deprivation levels.

## Methods/Design

### Design

Mortality followback postal survey with bereaved relatives of people who died from cancer, with a nested case-control study of home vs. hospital deaths, and informed by a pilot study [16].

Finding out the views of bereaved relatives is important for three main reasons. Firstly, because this is the only way to get a population-based perspective on end-of-life care, and an understanding of the use and experiences of care (with reasonable evidence that their evaluation of services matches those of patients)[17] relating to a consistent time period close to the patient's death (defined in this study as the three months before death). Secondly, relatives' views are important as research demonstrates their crucial role in caring and supporting patients to die at home [15]. Thirdly, relatives are often carers but also recipients of end-of-life care (which includes bereavement support); surveying bereaved relatives allows us to understand how well they cope with their loss and grief.

Notwithstanding, bereaved relatives are a vulnerable group and engaging in research can be distressing and difficult for them. Few would currently accept the position that bereavement research is unethical per se, particularly in light of research findings showing benefits as perceived by bereaved relatives[18-21]. These studies and our pilot study have indicated that most bereaved relatives welcome the opportunity to make a contribution towards improving care for others by taking part in research. Furthermore, using postal surveys of bereaved relatives is a well established and accepted method of research in end-of-life care, with tradition in the UK and indeed recommended in the recent National End of Life Care Strategy[14,22,23]. It has been shown that this kind of surveys can be carried out in an ethical manner. For these reasons, we have decided to conduct the study, conscious of the ethical challenges and committed to minimising the risks of harm and maximising the benefits for potential participants. We have reviewed the literature, consulted with experts in the field and conducted a careful pilot study with 20 bereaved relatives to decide on best design approaches.

A postal approach was chosen as the most appropriate and practical data collection method, given the large size of the study population and robust postal research methodologies with bereaved relatives developed in the UK. An RCT of postal vs. face-to-face methods using an adapted version of the same questionnaire found that response rates (52% vs. 56%) and respondents' characteristics did not differ significantly [23]. Although there were more missing data in the postal version, postal responders rated satisfaction and symptom control more moderately, which may suggest lesser influence of social desirability. Ethical issues were also taken into consideration as a postal method allows more privacy and less intrusion than face-to-face or telephone interviews.

The nested case-control study will identify people who die at home (defined as the "cases") and compare their past exposure to suspected risk factors (for dying at home) with that of patients who die in hospital (the "controls"). The definition of cases in relation to place of death is not as clear as in studies measuring disease frequency, where those affected by the disease are considered the cases. Our approach was therefore to take home deaths as the "cases", given that these are rarer than hospital deaths. The QUALYCARE case-control study will compare the care received, preferences and other risk factors for dying at home, rather than costs or outcomes (for which place of death is seen as a potential risk factor and not an outcome). Because the association will be determined for each individual case-control pair, the study will determine more accurately than cross-sectional studies the potential for a causal link between risk factors and death at home.

Finally, the study includes an economic evaluation of costs and consequences. This takes a health service perspective but voluntary sector costs and informal care are also valuated, at the market price that would have to be paid by the NHS to provide these. The cost component is the more exploratory part of the study, focused on estimation rather than hypothesis testing, as so little is known about the topic.

### Setting

The survey takes place in four Primary Care Trust (PCT) areas within London. London has the lowest home death rates of all nine strategic health authorities in England (18.5% in 2008) [24]. This is likely to reflect the influence of what is known to be the strongest determinant of death at home - hospital availability and use [15]. The home death rate for cancer-related deaths is slightly higher but also low (20%) and inner London variation has been reported, with deprivation playing a role [25]. The four PCT areas in the study have contrasting cancer home death rates and deprivation levels (within London).

### Participants

Bereaved relatives of people aged 18 years or over who died from cancer in a one year period (March 2009-March 2010), and who lived in one of the PCTs in study.

#### Identification and sampling

Bereaved relatives are identified from death registrations, as the people who registered a cancer-related death (informant). In a similar study across eight cancer networks in the UK (2002-03), this was found to be a spouse or relative in 93% of the cases [26]. This identification process is conducted in two waves (Nov 2009 and May 2010) in complete confidence by the Office for National Statistics (ONS).

In each wave, the ONS team first identifies all cases of death registered four to ten months prior to the date invitation letters are planned to be sent out; they then screen for further eligibility criteria (Table 2). There is no firm evidence of what is the most appropriate timeframe to approach bereaved relatives for research (with optimal balance between information accuracy and distress minimisation). A recent national survey in Italy found the best timing in terms of responses rates was four to six months after death [27]. We have established a minimum of four months after the death has been registered to contact the bereaved relatives, to avoid the period of more intense grieving. Previous studies have contacted people at a minimum of three months after death registration. However, results from our pilot study suggested this might still be too early for some. We have therefore taken a more conservative approach with view to minimise distress and potential harm.

**Table 2:** Eligibility criteria

**Inclusion criteria**
• deceased last resident in one of the four PCTs in study, as recorded in the death registration;
• date of registration of death within four to ten months before invitation letters for participation are sent out;
• deceased aged 18 and over at time of death;
• cancer (ICD-10 codes C00-D48) recorded as "underlying cause of death" or in the lowest completed cause of death line in the death certificate.

**Exclusion criteria**
• death registered by a coroner;
• place of death other than an NHS hospital, the deceased's own home, a hospice or a nursing home;
• place of death unknown.

After eligible people are identified, a sample is then drawn by the ONS team. This is stratified by PCT and place of death - 1) home, 2) hospice, 3) nursing home, and 4) NHS hospital. The sample in each PCT includes: all deaths that take place at home; all hospice deaths; all nursing home deaths; and a random sample of NHS hospital deaths.

#### Recruitment and consent

Invitation letters are sent by the ONS team to the informants in death registrations sampled for the study, inviting them to take part and explaining how they had been identified. Letters to potential participants in Wave 1 were sent in January 2010; letters for Wave 2 were sent in July 2010.

The invitation letter first apologises in advance for any distress caused. It then assures potential participants that their contact details were not released and that instead the ONS agreed to contact them on behalf of the research team for the purposes of the study. Each letter is enclosed with an information leaflet signed by the Principal Investigator and the Director of Public Health from the local Primary Care Trust (PCT), explaining why the work is important and how it will be used to help improve care (information leaflets available from study website at http://www.kcl.ac.uk/qualycare). Contact points are given if people wish to talk about the study. The information leaflet, the study questionnaire, a reply slip (for people to decline to take part if they wish so), two freepost envelopes (one for the reply slip to be sent to the ONS and one for the questionnaire to be sent to the research team) and a leaflet with information about bereavement (produced by the Royal College of Psychiatrists) are also enclosed.

Potential participants are invited to complete and return the completed questionnaire directly to the research team. This is taken as consent to participate. If they do not wish to take part in the study, they are asked to return the reply slip to the ONS. Upon receipt, they are flagged as non-participants and are not contacted again. Two reminders, with a further copy of the questionnaire, are sent by the ONS at two-weekly intervals to those who do not respond.

### Data collection

The data are collected at one point in time from each participant by a postal self-completed questionnaire. A follow-up telephone interview (of about 15 minutes) may also take place, although this is entirely optional, and only if participants write in the questionnaire they agree and provide their contact details. The interview aims to clarify information if appropriate (e.g. answers which appears to be accidentally missing) and to talk about the questionnaire, so the researcher ascertains the impact and effect of the questionnaire on the participant (to screen for distress and serious concerns). The period of data collection is planned to be eight months, including time for the second and last wave of questionnaires to be returned and follow-up telephone interviews to be made.

#### Questionnaire

The study uses an adapted short form of a questionnaire developed by Ann Cartwright in the late 1960's to measure patients' experiences of the last year of life from the perspective of their bereaved relatives [28]. Since then, versions of Cartwright's questionnaire have been used successfully in a number of surveys [26,27,29-32] We have integrated in it four brief and robust measurement tools previously used in cancer and end-of-life care studies (Table A in additional file 1). These tools collect information on health and social care services use and informal care (Client Service Receipt Inventory - CSRI) [33,34], patient palliative outcomes in the week prior to death (Palliative Outcome Scale - POS) [35,36], health-related quality of life (EuroQoL EQ-5D) [37], and respondents' bereavement outcomes (Texas Revised Inventory of Grief - TRIG). (38-40) Further questions explore preferences for place of death, relevant local issues and socio-demographic and clinical data. The format and navigation of the questionnaire was refined according to cognitive theory literature[41]. The resulting QUALYCARE questionnaire has been piloted and improved in a pilot study with 20 bereaved relatives, recruited via the palliative medicine department of a London hospital[16].

Completing the questionnaire takes about 60 minutes. Participants are given the contact details of the research team, should they need assistance.

### Practical and operational arrangements

Once identified, all sampled cases are assigned study identification (ID) numbers by the ONS team, who then provides the research team with a spreadsheet of these numbers (by PCT of residence, gender of the deceased and place of death coded as home, hospice, nursing home or hospital). The spreadsheet enables the ONS team and the research team to communicate using study ID numbers with a view to prepare the questionnaire packs, conduct the mailings and monitor recruitment. Questionnaire packs are prepared by the research team using the spreadsheet to personalised each pack by PCT and gender of the deceased (there are two versions of the questionnaire - one related to female deceased and one to male deceased). Questionnaires and reply slips are also marked with the respective study ID number. The packs are then delivered to the ONS, where address labels and personalised invitation letters are included and postage is organised. The research team manages the questionnaire returns and notifies the ONS of study ID numbers to prevent follow-up mailings to people who decline participation; ONS reports back to the research team all declinations to participate (made either by phone or post) with reasons if people volunteer them, as well as any complaints received.

Upon receipt of a completed questionnaire, the research team records arrival into the spreadsheet, checks completion, levels of distress and grief intensity, and safely store the questionnaire in a locked cabinet in a locked office in the team's department. Access to the data is restricted and controlled. If there is need for clarifications regarding the questionnaire or follow-up to assess the impact of the questionnaire on the participant and potential distress, and provided the participant has agreed to be contacted and provided contact details, a member of the research team makes contact to arrange a telephone interview.

At the end of each wave, the ONS provides the research team with an anonymised and encrypted dataset of all eligible cases of deaths in each of the PCTs (including participants and non-participants) with individual information on study ID numbers, PCT of residence, age, date of death occurrence, date of death registration, underlying cause of death, other causes of death, place of death, gender, place of birth, electoral ward of last residence of the deceased and their relationship to the informant. These data are important to understand what population is excluded from the study and who decides not to take part. All information is treated confidentially, following ONS regulations and the data management guidelines of the research team's department. The study has been approved by an NHS Research Ethics Committee (REC ref no.: 09/H0808/85).

### Procedures to identifying and handling distress

Receiving the invitation letter and completing the questionnaire can lead to a reawakening of bereavement and any associated distress or anger. It can also lead to disclosure of information by the participants which may require follow-up action by the research team. For example, it may prompt unresolved issues about bereavement and grief in the participant. Published guidelines by Colin Murray Parkes on how to conduct ethical bereavement research are being followed [42] and a series of measures are in place to identify and deal with any distress, complaints and sensitive issues arising in the course of the study (Table 3).

**Table 3:** Measures for handling distress and sensitive issues

**1. Written protocol for dealing with queries and distress**
This was made available to those involved in information giving and interviewing (members of the research team, the PI Department's administrative team, the ONS team) so they carry out these tasks sensitively, with attention to any distress (potential or real), and handling this appropriately;

**2. Communication log record**
Conversations maintained with people approached for the study (or with anyone on their behalf) or with individuals requesting for information about the study (e.g. via website feedback form) are recorded to facilitate review, as need be;

**3. Screening for need to follow-up**
A member of the research team reads and checks the questionnaire soon after its arrival, particularly the responses to questions on grief intensity, to screen for cases which may require follow-up action. Should the participant agree to be contacted and write down in the questionnaire her/his contact details, the researcher makes contact to ascertain the impact and effect of the questionnaire on the participant, and to direct to sources of help if appropriate. The researcher approaches this with sensitivity and handles any distress appropriately.

◦ If specific concerns arise, the researcher informs the participant about sources of local support and asks the participant if s/he wishes to be given the contact details or if he/she would like the researcher to contact local services for contacts/visits to be arranged;

◦ If the researcher has a high level of concern about a participant (e.g. if the researcher suspects a participant is very depressed and possibly suicidal) this is discussed as a matter of urgency with the PI, who is a Consultant in Palliative Medicine with extensive clinical experience of working with distressed family members and addressing their distress, to proceed with appropriate action to further assist and aid the participant in question, if this is felt appropriate. Complaints are regarded as situations of high level of concern and treated in the same manner.

### Data entry

Answers to the questionnaire are entered into a pre-designed SPSS database (version 17.0). This excludes any personal information that might have been provided by the participant, such as contact details; these are kept only in the paper questionnaire, which is securely stored in a locked cabinet. Data entry is continuously monitored through supervision meetings where a set of rules develops and exceptions are discussed. A decision diary with rules, exceptions and decisions constructs an audit trail to enable transparency. Ten percent of the data are double-entered and cross-checks identify the percentage of items with discordances, missing data and systematic errors (percentage of double-checked data to be adjusted according to outcome of cross-checks). Once completed and checked, the questionnaire database will then link with the anonymised dataset provided by the ONS (using study ID numbers).

### Data analysis

Numbers of all cancer deaths, participants and non-participants, including those ineligible and those who decline to participate or do not reply will be reported in a flowchart. Participants and non-participants will be compared in terms of clinical and socio-demographic variables of both the deceased and their relatives, to investigate possible selection bias. The influence of place of death and timing from death to first contact will also be investigated. T-tests will be used to compare continuous data which are normally distributed, Mann-Whitney tests will be used with non-parametric continuous or ordinal data, and χ^2 ^tests will be used with categorical variables (significance level set at 0.05).

#### Place of death and place of end-of-life care

The data allow reporting the place of death for both participants and non-participants. For participants, comparisons will be made between place of death as reported in the death registrations and as reported by the bereaved relatives, examining percentage and type of disagreements. Place of death will also be compared with the place where the patients spent most part of the three months before death, as reported by their relatives (objective 1.1 in Table 1).

#### Descriptive and bivariate analysis of home, hospice, nursing home, and hospital deaths

Frequency tables and descriptive statistics will be used to compare home with hospital, hospice and nursing home deaths in terms of preferences for place of death (objective 1.2) - of patients and of their relatives, and for the latter at three months prior to death, changes in the three months before death (reported retrospectively) and post-death. Numbers and percentages (with 95% CIs) where the patient and the relative disagreed regarding place of death and the type of disagreements will also be reported. χ^2 ^tests will be used to test for differences.

Frequency tables and descriptive statistics will report outcomes by place of death (objective 1.3). These will show numbers and percentages of patients who experienced pain, anxiety/depression, confusion, and other symptoms, and more severe levels of each symptom in the week of death. Symptom scores in the POS and EQ-5 D will be compared for pain and anxiety/depression and percentage and type of disagreements reported. We will also compare item responses and total scores in the POS, EQ-5 D and TRIG, satisfaction with the different types of formal care (both as categorical - to identify those experiencing poor care - and ordinal data - very poor/poor/fair/good/very good/excellent) and attainment of preferred place of death for patients and relatives, between home and hospital, hospice and nursing home deaths. Differences between home and hospital, hospice or nursing home deaths will be tested using independent-samples t-tests to compare normally distributed continuous data, Mann-Whitney tests to compare non-parametric continuous or ordinal data, and χ^2 ^tests to compare categorical variables.

Frequency tables and descriptive statistics with numbers and percentages (95% CIs), medians (with IQ ranges) or means (with SDs) will report the use of informal and formal care in the three months before death, by place of death (objectives 1.4 and 1.5). Use will be examined both as categorical (yes/no) and ordinal or continuous data (median/mean depending on distribution normality of hours of care, numbers of visits and admissions). Home deaths will be compared with the three other places of deaths (one-to-one) using independent-samples t-tests to compare normally distributed continuous data, Mann-Whitney tests to compare non-parametric continuous or ordinal data, and χ^2 ^tests to compare categorical variables. Continuous data on service use are expected to be skewed but we will examine histograms and box plots to confirm this.

Data on service use in the last three months before death, collected using the CSRI, will be combined with appropriate unit costs to calculate service costs. Unit costs will be mostly obtained from the 2009 Unit Costs for Health and Social Care publication[43]. We plan to cost the informal care by using the hourly wage rate of a formal caregiver that would have to be paid to replace informal care if this was not available (market price method). Sensitivity analyses will be carried out by calculating informal care costs using a lost productivity method (time lost from work, i.e. human capital approach). We will compute informal and formal care costs, and total costs. The cost data are expected to be skewed but we will examine histograms and box plots to confirm this. Depending on normality, we will report mean or median costs per person per day and mean or median total costs per person in the three months before death. Differences between home and hospital, hospice and nursing home deaths will be tested using independent-samples t-tests or Mann-Whitney tests. We will explore the relationship between costs and outcomes in the different settings.

Similar analyses will be conducted to compare home vs. institutional deaths (merging hospital, hospice, nursing home deaths) (objective 2), to compare areas with high vs. low home death rates and areas with high vs. low deprivation levels (objective 3).

#### Multivariate analysis of factors influencing death at home

Following bivariate analyses comparing home with other places of death, logistic regressions will be conducted to determine the relative influence of different factors on death at home (vs. hospital death and vs. institutional death), place of death being the dependent categorical variable (home - yes/no) (objective 4). Potential predictors of death at home identified in bivariate analyses will be entered in the regression. These will be grouped in three categories - illness related, personal (including preferences), and environmental factors (including care components), following a model of factors influencing death at home in cancer [15]. Odds ratios with 95% CIs will be calculated for each factor and compared to identify the strongest and most significant. Results will be compared with the bivariate analysis to identify factors that lose significance or strength to others. The adjusted R^2 ^statistic will be calculated to determine the amount of variance accounted for in the models.

Regression analyses will be performed for areas with high and low home death rates separately (sub-group analysis) and results will be compared in terms of factors retained in the models and those that lose significance or strength to others, and the amount of variance explained. In the same way, regressions will also be performed within areas with high and low social deprivation levels.

#### Multivariate analysis of the influence of death at home on outcomes and costs

Regression analyses will be conducted to determine the relative influence of death at home (vs. hospital death and vs. institutional death) on the costs (informal, formal and total) and on each outcome (dependent variables), controlling for other factors (objective 5). First, bivariate analyses will be undertaken to identify predictors of each of the palliative outcomes and cost-drivers, other than death at home; these will be entered in the regression, alongside death at home. Different multivariate approaches will be undertaken including logistic regression and linear regression. We plan to use bootstrapping with cost data as these are expected to be skewed, to generate more robust 95% CIs;[44] results will be compared to identify the best model.

The odds ratios and 95% CIs for death at home will be compared with those for other factors, to assess its strength and significance relative to others. Results will be compared with bivariate analyses to examine whether death at home lost or gain significance or strength. Similar regression analyses will be performed for areas with high and low home death rates separately (sub-group analysis) and results will be compared in terms of the influence of death at home. Regressions will also be performed within areas with high and low social deprivation levels.

### Power calculation

Sample size calculations were carried out following Altman's methods[45] for the main component of the analysis which will compare home deaths with institutional deaths, to enable powered comparisons in terms of the preferences for place of death and the use of and satisfaction with home care. Calculations were based on estimates from a London survey (1995-96) which also used an adaptation of Cartwright's questionnaire and compared home deaths vs. those taking place elsewhere for cancer patients [30]. Three variables were examined: patient preference for death at home (yes/no), help of community nurse (yes/no), and satisfaction with GP care (poor vs. fair/good/excellent). Standardised differences in proportions were 0.11 in deceased who preferred to die at home, 0.72 in people who had help from community nursing, and 0.22 in people dissatisfied with GP care. Applying these to Altman's nomogram (power of 80%, significance of 0.05), it was estimated that a sample of 30 would be sufficient to detect differences in preferences of the same magnitude as in the previous London survey, 60 would allow detection of differences in the use of community nursing but a much bigger sample size 800 would be needed to detect the same differences in satisfaction with GP care (0.11 amongst home deaths and 0.19 amongst deaths elsewhere were dissatisfied with GP care, with a trend towards significance in the previous study). On balance, we felt that the difference in GP care was small and did not justify the increase in sample size. Therefore, it was decided to aim for a minimum standardised difference of 0.30 which will be detectable with a minimum sample of 350 bereaved relatives, split into home, hospital and hospice/nursing home, home deaths and institutional deaths for the main component of analysis and into home and hospital deaths for the case-control analysis. A sample of 350 will also accommodate logistic and linear regression analyses with a maximum of 35 variables entered and 19 retained, if the rules of thumb suggested by Altman apply (n/10 variables and square root of sample size respectively) [45].

Lack of data on the magnitude of differences on patients and relatives' outcomes and costs precluded further calculations. Measuring the costs of care usually requires relatively large sample sizes due to high variance in costs (large SDs)[46] but the cost component is the more exploratory part of the analysis and will be focused on estimation rather than hypothesis testing, as so little is known about this. Post-hoc calculations will be performed at the end of the study to determine the power and significance of the findings.

Based on responses achieved in a recent survey, we have accommodated a scenario of 38% response rate with an extra 10% of missing data [26]. To get a sample of ~350 completed questionnaires for the most detailed level of analysis (case-control of home vs. hospital deaths) we estimated we need to sample ~1,450 deaths over a one year period. Data on numbers of cancer deaths and of home deaths in each of the selected PCTs provided by the Thames Cancer Registry [47], applied against NHS acute hospital and hospice death rates and nursing home death rates in the respective Cancer Networks(26;48) suggested our estimated sample was feasible, although temporal fluctuations were expected (the study takes place in 2009/10).

Based on the estimated figures by place of death, a sampling strategy was drawn. We decided to include all eligible home deaths, hospice deaths and nursing home deaths in all four PCTs, and a random sample of 150 NHS hospital deaths in each PCT. The number of people to be sent questionnaires was projected to approximate 1,450, of which we expected ~550 returned questionnaires, ~500 with complete data.

### Interim analyses and stopping rules

Prior to commencing the study, it was decided that if response rates were lower than 30% or if there were serious complaints that could not be addressed, we would stop the study. Results from Wave 1 were reviewed with the project steering group in March 2010. The response rate achieved by then was higher than 40% and there were no serious complaints that could not be addressed. Therefore, the study progressed to Wave 2.

### Project steering group

This consists of an independent group responsible for data-monitoring. The group comprises representatives of the ONS, the PCTs in the study, local palliative care services, the Department of Heath End-of-Life Care Team, and a user representative (bereaved relative). Progress is reported to the group every three months.

## Discussion

The QUALYCARE study faces two main challenges. First, ethical and data protection issues and practicalities are prominent in the study and have strongly determined its design. The sensitivity of the topic requires complicated procedures, compassion and attention to detail from all the staff involved. Secondly, there is a lack of evidence in the emerging area of cost measurement at the end of life to inform study design and procedures, thus methods and conclusions relating to costs will be in essence exploratory. Recall bias and use of caregivers as proxies for evaluating service use and palliative outcomes are important aspects to consider when interpreting the data [17,36].

Despite limitations, the QUALYCARE study is one of the most detailed population-based surveys of our time about the end-of-life care provided to people with cancer. Being carefully designed to be ethically and scientifically sound, it contributes to advancing the science in end-of-life care research. Case-control studies such as the one nested in QUALYCARE are rare in end-of-life care research[49] despite offering a strong alternative to longitudinal studies (which pose numerous practical, ethical and methodological challenges in this field) for studying natural variation in outcomes. Our health economic evaluation component will add to pioneering stages in the methodological development of cost measurement at the end of life.

More importantly, the QUALYCARE study could discover variations in the underlying individual reality of place of death for people dying from cancer and their families. This may include differences in the preferences, in palliative outcomes for patients, in bereavement outcomes for relatives, and in the costs of caring for people dying at home as compared to institutional settings, not just for the health care system but for families. Our findings will, we hope, help improve care for cancer patients and their families locally, and in comparable settings across the UK and beyond.

## List of abbreviations

CI: Confidence Interval; CSRI: Client Service Receipt Inventory; GP: General Practitioner; IQ: Inter-Quartile; NHS: National Health Service; ONS: Office for National Statistics; PCT: Primary Care Trust; POS: Palliative Outcome Scale; SD: Standard Deviation; TRIG: Texas Revised Inventory of Grief

## Competing interests

The authors have received funding in the past five years from the Department of Health (partner in the study) for conducting end-of-life care research and, although unlikely, it is possible this organisation may gain or lose financially from the publication of this manuscript. The authors declare that they have no other competing interests.

## Authors' contributions

BG and IJH conceived the study, and PMcC, SH and JK made substantial contributions to its conception and design. BG and IJH negotiated the collaboration and acquisition of anonymised mortality data from the Office for National Statistics. BG coordinates the study fieldwork including sampling, data collection and data entry, supported by IJH, JK and SH. All authors are expected to make substantial contributions to the analysis and interpretation of the data. BG drafted this manuscript and PMcC, SH, JK and IJH revised it for important intellectual content. All authors read and approved the final manuscript.

## Pre-publication history

The pre-publication history for this paper can be accessed here:

http://www.biomedcentral.com/1471-2407/10/400/prepub

## Supplementary Material

Additional file 1 **Table A**. Click here for file
