# Supplementary material for: Variations in the quality and costs of end-of-life care, preferences and palliative outcomes for cancer patients by place of death: the QUALYCARE study
Source: BMC Cancer. 2010 Aug 2;10:400. doi: 10.1186/1471-2407-10-400 (PMC2919503; doi:10.1186/1471-2407-10-400)
Supplement: Additional file 1 — Table A. [file 1471-2407-10-400-S1.DOC]

**Table A**. Composition of the QUALYCARE questionnaire

| **Part** | **Tools and content** |
| --- | --- |
| Part 1  The care received in the last 3 months of life | This first part of the questionnaire uses the *Client Receipt Service Inventory* (CSRI) to collect information on health and social care services and informal care provided in the three months before death, and relatives’ days off work (in the three months prior and after death). The CSRI was developed by the Personal Social Services Research Unit (University of Kent) for use in mental health service economic evaluations, takes about 20 minutes to complete and has been used nationally and internationally in over 200 studies, including end-of-life care studies.(1;2) Recent comparisons of the CSRI with data records in primary care found a good level of agreement between the number of GP visits recorded in case records and CSRI postal responses.(3) Service use data are combined with unit cost information (much of which is also available from the Personal Social Services Research Unit) to calculate service costs from a bottom-up approach. |
| Part 2  Living circumstances and your views on the care | Part 2 derives from the original and well-established questionnaire developed by Ann Cartwright in the UK in the late 1960’s.(4) Cartwright’s study was about the last year before death of a nationally representative sample of people who died in England; her questionnaire was designed to capture bereaved relatives’ satisfaction with various aspects of the care received in the year before death. Short versions of the questionnaire including postal forms have been developed and used in the UK and internationally in various settings and populations, including relatives of cancer patients.(5-10) Qualitative work led to refinements (into what is known as the VOICES questionnaire) and most recently a psychometric validation study showed encouraging results on the reliability and discriminatory power of the questions.(11) We have used a short version of the questionnaire adapted to the purposes of the QUALYCARE study, and revised it during and after the pilot study.(12) We have organised this part 2 into sections (care at home, in hospital, in hospice and in a care home, and a final section on the existence of a key professional contact during the three months before death, and awareness/ discussion of prognosis). Each of the sections ends with a free-text comment box. |
| Part 3  The last week before he/she died | This part includes the *Palliative Outcome Scale* (POS) as a measure of patient outcomes in the week of death, perceived by the bereaved relatives. This is a short easy-to-use clinical outcome measure originally developed and validated in eight end of life and palliative care settings in the UK, including hospital, community, in-patient hospice, outpatient, day care and general practice.(13) The POS was based on a systematic literature review of existing scales and it has ten items including physical and psychological symptoms, information and spiritual needs, family and practical problems plus an open field for additional reports. It has been designed to reflect patient-centred care and addresses problems for patients and families as critical to quality care. Recent comparisons of patient and caregiver POS ratings showed substantial agreement for pain, moderate for four items, and fair for three and slight for two.(14) The POS is followed by part 1 of the EuroQoL EQ-5D, which is a generic preference-based measure of health status/health related quality of life, applicable to a wide range of health conditions including cancer and increasingly used in economic evaluations. Part 1 of the EQ-5D provides a simple descriptive profile and an index value for health status, is ideally suited for use in postal surveys and can be used by proxies (in this case, it is completed retrospectively by bereaved relatives relating to the patients’ health status/health related quality of life in the week before death). Part 1 of the EQ-5D scores ‘none’, ‘some’ or ‘extreme’ problems in each of the five quality-of-life domains, namely mobility, self care, usual activities, pain/discomfort and anxiety/depression. The EQ-5D was chosen as people find it easy to complete and it has been validated in advanced cancer, with findings showing that it is valid, reliable and responsive to change over time, and can serve as an outcome measure in end-of-life care settings.(15) We have used the EQ-5D to measure health status in the last week of life but also asked respondents to answer the same questions looking back at three months before death, to identify changes. |
| Part 4  Circumstances of death and personal preferences | This part includes questions on the actual place of death, patients’ preference for place of death (as perceived by the relatives), discussion of preferences with professionals and relatives, relatives’ preference (looking back at three months before death, changes in the three months before death, what they would prefer if they were to choose again, and if all things were in place), family presence at death, a question on patient feeling at peace in the week before death, and a final question on whether there were any aspects of care people felt unhappy with, and if any action was taken. |
| Part 5  You and how you feel | Part 5 includes the *Texas Revised Inventory of Grief* (TRIG), a measure of the intensity of grief experienced by bereaved people. This is a 21-item assessment tool which is easily and rapidly done with a minimum of intrusion into the bereaved person’s already disrupted life.(16) The items have been developed using factor analysis and the resulting scale has demonstrated reliability, construct and discriminant validity. TRIG has been successfully used clinically and in studies with bereaved relatives of cancer patients (17;18) and in our pilot study. This part ends with a question on whether bereavement support has been used and if so how helpful it was. It also refers people who may want more information on bereavement and sources of support to the bereavement leaflet enclosed. |
| Part 6  Final questions and experience of the questionnaire | Part 6 asks general socio-demographic questions (gender, age, religion, ethnicity), participant’s views on the questionnaire (distressing, helpful), whether they are willing to be contacted by the research team and if they wish to receive a brief summary of the findings. It ends with two blank pages in case participants wish to write anything else about the care received or care they would have liked to receive. |

**References**

(1) Beecham J, Knapp M: **Costing psychiatric interventions**. In: *Measuring mental health needs*. 2^nd^ Edition. Edited by Thornicroft G. London: Gaskell; 2001.

(2) McCrone P. **Capturing the costs of end-of-life care: comparisons of multiple sclerosis, Parkinson's disease, and dementia**. *Journal of Pain & Symptom Management* 2009;**38**(1):62-7.

(3) Patel A, Rendu A, Moran P, Leese M, Mann A, Knapp M. **A comparison of two methods of collecting economic data in primary care**. *Family Practice* 2005;**22**(3):323-7.

(4) Cartwright A, Hockey L, Anderson JL: *Life before death*. London: Routledge and Kegan Paul; 1973.

(5) Addington-Hall J, McCarthy M. **Dying from cancer: results of a national population-based investigation**. *Palliative Medicine* 1995;**9**(4):295-305.

(6) Addington-Hall J, Shipman C, Burt J, Ream E, Beynon T, Richardson A: *Evaluation of the education and support programme for district and community nurses in the principles and practices of palliative care*. London: King's College London; 2006.

(7) Costantini M, Beccaro M, Merlo F, ISDOC Study Group. **The last three months of life of Italian cancer patients. Methods, sample characteristics and response rate of the Italian Survey of the Dying of Cancer (ISDOC)**. *Palliative Medicine* 2005;**19**(8):628-38.

(8) Karlsen S, Addington-Hall J. **How do cancer patients who die at home differ from those who die elsewhere?** *Palliative Medicine* 1998;**12**(4):279-86.

(9) Koffman J, Higginson IJ. **Accounts of carers' satisfaction with health care at the end of life: a comparison of first generation black Caribbeans and white patients with advanced disease**. *Palliative Medicine* 2001*;***15**(4):337-45.

(10) Lecouturier J, Jacoby A, Bradshaw C, Lovel T, Eccles M. **Lay carers' satisfaction with community palliative care: results of a postal survey. South Tyneside MAAG Palliative Care Study Group.** *Palliative Medicine* 1999;**13**(4):275-83.

(11) Jacoby A, Lecouturier J, Bradshaw C, Lovel T, Eccles M. **Feasibility of using postal questionnaires to examine carer satisfaction with palliative care: A methodological assessment**. *Palliative Medicine* 1999;13(4):285-98.

(12) Higginson IJ, Hall S, Koffman J, Riley J, Gomes B. **Time to get it right: are preferences for place of death more stable than we think?** *Palliative Medicine* 2010;**24**(3):352-3.

(13) Hearn J, Higginson IJ. **Development and validation of a core outcome measure for palliative care: the palliative care outcome scale. Palliative Care Core Audit Project Advisory Group**. *Quality in Health Care* 1999;**8**(4):219-27.

(14) Higginson IJ, Gao W. **Caregiver assessment of patients with advanced cancer: concordance with patients, effect of burden and positivity**. *Health & Quality of Life Outcomes* 2008;**6**:42.

(15) Xia W, Hwang SS, Chang VT, Osenenko P, Alejandro Y, Yan H, et al. **Validity, reliability and responsiveness of Euroqol (EQ5D) in patients (Pts) receiving palliative care (PC) [abstract]**. *Journal of Clinical Oncology* 2005;**22** (Supplement 16): 8082.

(16) Faschingbauer TR, Zisook S, DeVaul R: **The Texas Revised Inventory of Grief**. In: *Biopsychosocial aspects of bereavement.* Edited by Zisook S.Washington D.C.: American Psychiatric Press, Inc.; 1987:109-24.

(17) Grande GE, Ewing G. **Informal carer bereavement outcome: relation to quality of end of life support and achievement of preferred place of death**. *Palliative Medicine* 2009;**23**:248-56.

(18) Ringdal G, Jordhoy MS, Ringdal K, Kaasa S. **Factors affecting grief reactions in close family members to individuals who have died of cancer.** *Journal of Pain and Symptom Management* 2001;**22**(6):1016-26.
